# Efficacy of 2-undecanol produced by *Paenibacillus polymyxa* KM2501-1 in controlling *Meloidogyne incognita*

**DOI:** 10.1128/spectrum.03062-24

**Published:** 2025-06-26

**Authors:** Fan Yang, Wei Dai, Hua Xue, Wen Chen, Chen Liu, Yaru Tian, Wanli Cheng, Jibin Zhang

**Affiliations:** 1 National Key Laboratory of Agricultural Microbiology, College of Life Science and Technology, National Engineering Research Center of Microbial Pesticides, Huazhong Agriculture University 124443, Wuhan, Hubei, China; 2 State Key Laboratory of Biocatalysis and Enzyme Engineering, School of Life Science, Hubei University 12563, Wuhan, Hubei, China; USDA-ARS San Joaquin Valley Agricultural Sciences Center, Parlier, California, USA

**Keywords:** *Paenibacillus polymyxa*, 2-undecanol, *Meloidogyne incognita*, root exudate, metabolomics analysis

## Abstract

Root-knot nematodes (RKNs) are notoriously challenging to control because of their broad host spectrum and swift proliferation. Consequently, there is an urgent need to develop safe and effective nematicides. Microorganisms and their metabolites have the potential to control RKNs safely and effectively. *Paenibacillus polymyxa* KM2501-1 can produce a range of volatile organic compounds (VOCs) with nematicidal activities, among which 2-undecanol is a potential biocontrol agent against *Meloidogyne incognita*. Nonetheless, the mechanism by which 2-undecanol prevents RKN infection in plant roots remains unclear. 2-Undecanol has many activities against *M. incognita in vitro*, including contact (LC_50_ = 34.5 mg/L), fumigation (LC_50_ = 191.6 mg/L), attraction, locomotion inhibition (frequency of head thrash and body bend reduced by 84.0% and 97.0% at concentration of 40 mg/L) and egg hatching inhibition (by 98.5% at concentration of 80 mg/L). Upon treatment with 2-undecanol, a noticeable reduction in the number of galls was observed, and the control efficacy reached 60.8% at the dose of 5 mg/pot. Metabolomics analysis revealed that the application of 2-undecanol significantly influenced the composition of root exudates of tomato. Among the 17 differential metabolites, the up-regulated 10-undecenal could attract and kill *M. incognita* J2 larvae at a concentration of 100 mg/L, and cyclohexylamine had nematicidal activity at the concentration of 1,000 mg/L. This study revealed the mechanism of 2-undecanol inducing plant roots to secrete nematicidal substances with attractant activity, effectively controlling RKNs, which could pave the way for the development of environmentally friendly and cost-effective nematicides.

**IMPORTANCE:**

Root-knot nematodes (RKNs) pose a formidable challenge in management due to their extensive host range and rapid reproductive capacity. Microorganisms and their metabolites hold the promise of safely and effectively controlling RKNs. 2-Undecanol, produced by *Paenibacillus polymyxa* KM2501-1, is a potential biocontrol agent against *Meloidogyne incognita*. However, the mechanism of how 2-undecanol prevents RKN infection in plant roots remains unclear. We further revealed the increase in the abundance of root exudates with nematode-attractive and nematocidal activity induced by 2-undecanol in plants. This study highlights the important role of 2-undecanol produced by *P. polymyxa* KM2501-1 against nematodes, which lays a theoretical foundation for the development of *P. polymyxa* KM2501-1 as a new microbial nematicide while 2-undecanol is a new microbial-derived nematicide.

## INTRODUCTION

Plant parasitic nematodes (PPNs) are endoparasites that trigger a wide range of soil-borne diseases globally, leading to an estimated economic loss of 150 billion dollars annually. Among these, root-knot nematodes (RKNs; *Meloidogyne* spp.) have been particularly devastating for the economy and agricultural productivity (1, 2). There are numerous species of RKNs, with approximately 100 species documented to date. Four species within this group cause the majority of agricultural nematode diseases, with *Meloidogyne incognita* being the most serious and widely distributed species in China (2, 3).

RKNs undergo a multiple-stage lifestyle; they start as eggs. Within the eggs, they develop into first-stage juveniles, then hatch into infectious second-stage juveniles. The second-stage larvae further develop into third- and fourth-stage larvae before maturing into adults, subsequently lay eggs, thereby completing the life cycle in a cyclical manner (4). Their remarkable adaptability, coupled with the short life cycle (approximately 4–8 weeks) and robust reproductive capability, can lead to significant damage to plants within a short timeframe, which makes their control even more challenging (5, 6).

Currently, chemical nematicides are commonly used for nematode control in crop production (7). In order to enhance control efficacy, the doses applied in practice often exceed the recommended levels (8). However, chemical control not only affects the quality of agricultural products and biodiversity, but also causes irreversible and severe environmental pollution (9, 10). Biological control can help reduce the use of chemical nematicides, and it is imperative that we take action to adopt biological nematicide alternatives (11, 12). Microbial agents can directly kill nematodes, indirectly control nematodes by producing certain metabolites that kill nematodes or trigger the host plant immune response, or affect the composition of plant root exudates, these pathways interconnect and work together (13, 14). Plants secrete substantial amounts of substances into the rhizosphere (15), significant changes in root exudates may affect the interactions of organisms within the rhizosphere, such as reducing the infection of pathogens (16, 17). Control efficacy of microorganisms on RKNs is highly susceptible to the interference of various external environmental factors. To overcome this problem, many efforts have been made to develop products using secondary metabolites of natural enemies of nematodes as biological control agents (18). Currently, some microbial and plant metabolites with significant effects on RKNs have been progressively identified, with avermectin being among the most crucial. Avermectins, a group of 16-membered macrocyclic lactones, are used as an antibiotic, insecticidal, acaricidal, and nematicidal agent produced by fermentation of *Streptomyces avermitilis* isolated from soil (19). Avermectin is an insect nerve agent with a broad spectrum, high efficiency, low residue, and safety to humans, livestock, and the environment (20). With the increasing use of avermectin, drug resistance has gradually developed in target organisms (21), and new nematicides need to be developed urgently.

*Paenibacillus polymyxa*, a model species of the genus *Paenibacillus* (22), is a common soil bacterium and a plant growth-promoting rhizobacteria. *P. polymyxa* produces a rich variety of metabolites, including antimicrobial peptides, lipopeptides, proteins, enzymes, exopolysaccharides (23–28), and volatile organic compounds (VOCs). VOCs produced include organic compounds such as aromatics, ketones, alcohols, and ethers, some of which have special biological activities such as bacteriostatic and nematicidal, or inducing systemic resistance in plants (29–31). They can interact with isolated organisms through the cracks in the soil (32). We previously isolated *P. polymyxa* KM2501-1, and its fermentation supernatant showed good contact and fumigation activities against *M. incognita in vitro*. It was determined that it could produce a variety of nematicidal VOCs by solid phase microextraction-gas chromatography-mass spectrum, among which 2-undecanol showed strong nematicidal activity and was a potential biocontrol agent for RKNs (33). In this study, the inhibitory activity of 2-undecanol against *M. incognita* was tested *in vitro,* and the control effect was evaluated in the pot experiment. Variations in root exudates from tomato plants following treatment with 2-undecanol were analyzed by metabolomics, and the inhibitory activity of several differential exudates against *M. incognita in vitro* was also tested. The aim of this study was to clarify that 2-undecanol, a metabolite of *P. polymyxa* KM2501-1, not only kills RKNs directly, but also affects root exudates to resist nematode infestation.

## RESULTS

### 2-Undecanol exhibits multiple inhibitory activities against *M. incognita in vitro*

**Fig 1:**
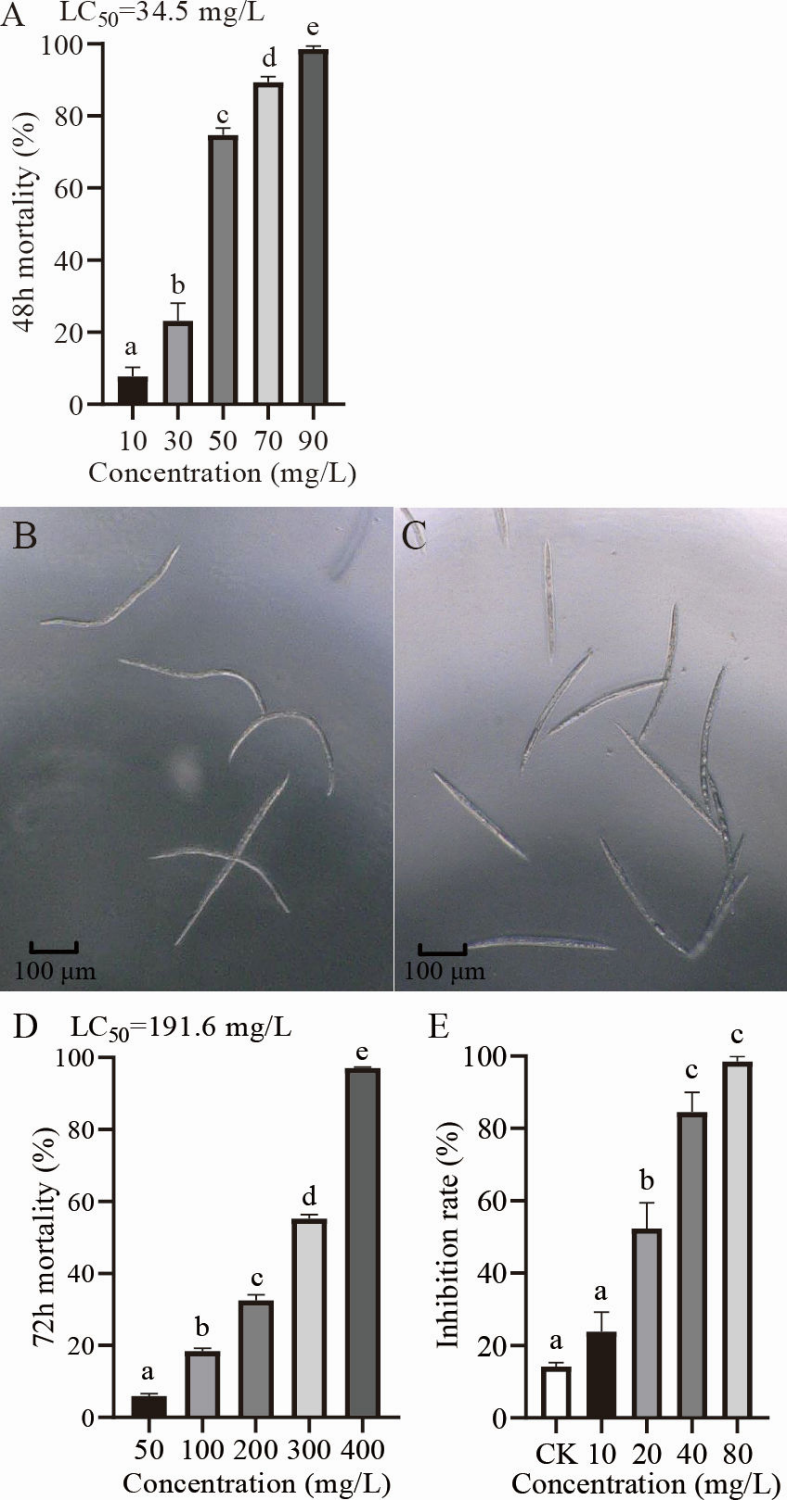
Nematicidal activity against *M. incognita* of 2-undecanol *in vitro*. (**A**) Contact activity against J2s of *M. incognita* immersed in solutions of 2-undecanol after 48 h. The mortality rates have been corrected by eliminating the natural deaths in the negative control according to the Schneider-Orelli formula. (**B**) The morphology of *M. incognita* disposed by the control group. (**C**) The morphology of *M. incognita* disposed by 2-undecanol at a concentration of 90 mg/L. (**D**) Fumigant activity against J2s of *M. incognita* of 2-undecanol after 72 h. The mortality rates have been corrected by eliminating the natural deaths in the negative control according to the Schneider-Orelli formula. (**E**) Effects of 2-undecanol on eggs hatching of *M. incognita* after 7 days. Data are shown as mean ± SE (*n* ≥ 3). Different lowercase letters indicate significant differences among treatments by the least significant difference (LSD) test (*P* < 0.05).

The contact activity of 2-undecanol was tested. It was found that 2-undecanol at 10–90 mg/L has nematicidal activity (Fig. 1A), and different concentrations of 2-undecanol can cause the death of *M. incognita* to a certain extent after 48 h contact with nematodes. The half-lethal concentration (LC_50_) of 2-undecanol for contact activity against *M. incognita* was approximately 34.5 mg/L. As shown in Fig. 1B and C, the nematodes in the control group are actively wriggling, while those disposed with 2-undecanol are rigid and dead. The fumigation activity of 2-undecanol against *M. incognita* was also determined (Fig. 1D). Different concentrations of 2-undecanol can lead to the death of nematodes in a concentration-dependent manner without contact with nematodes. The LC_50_ of 2-undecanol fumigation was approximately 191.6 mg/L. The results of contact and fumigation activity are consistent with the findings of the previous study (33).

Chemotactic model was used to detect the chemotactic activity of 2-undecanol to *M. incognita*. The results showed that 2-undecanol had attractant activity to nematodes, in a concentration-dependent manner, at the concentrations of 100, 1,000, and 10,000 mg/L (Table 1). The attractant activity increased with the increase in concentration. When the concentration was 100 mg/L, the chemotaxis index (C.I.) was 0.091, showing weak attractant activity. When the concentration was 1,000 mg/L and 10,000 mg/L, the C.I. was above 0.2, which showed very significant attractant activity compared with the control.

**TABLE 1:** J2s of *M. incognita* chemotactic response to 2-undecanol^*a*^

2-Undecanol concentration (mg/L)	Chemotaxis index (C.I.)
0	0.008 ± 0.026^b^
100	0.091 ± 0.019^c^
1,000	0.224 ± 0.027^d^
10,000	0.229 ± 0.035^d^

^*a*^ The data are shown as the mean ± SE (*n* ≥ 5). Different lowercase letters indicate significant differences among treatments by the LSD test (*P* < 0.05).

Head thrashing is one of the most basic and simple movement modes of nematodes. Nematodes usually move by passing their bodies forward or backward along a sinusoidal movement. A sinusoidal movement produced by a nematode represents the bending of its body once. Head thrashing can occur independently or when the whole body is bent. The frequency of head thrash and body bend can reflect the strength of the locomotion ability of the nematode (34, 35). In order to investigate the effect of 2-undecanol on the locomotion of *M. incognita*, the frequency of head thrashing and body bending of J2s treated for 24 h was observed. When the concentration was 10 mg/L, the motor behavior was significantly inhibited compared with the control, and the frequency of head thrashing and body bending decreased from 25.00 and 10.00 to 17.00 and 7.00, respectively (Table 2). When the concentration reached 40 mg/L, although the nematode did not die, the frequency of head thrashing and body bending decreased to 4.00 and 0.30, respectively, and the nematodes were basically unable to move. The results showed that 2-undecanol inhibited the locomotion of nematodes in a concentration-dependent manner.

**TABLE 2:** Effects of 2-undecanol exposure on head thrash and body bend exposures were performed from *M. incognita^a^*

Concentration (mg/L)	Head thrash (Hz)	Body bend (Hz)
0	25.00 ± 1.15^b^	10.00 ± 0.99^b^
10	17.00 ± 1.23^c^	7.00 ± 0.57^c^
20	11.00 ± 0.91^d^	4.00 ± 0.53^d^
40	4.00 ± 0.51^e^	0.30 ± 0.14^e^

^*a*^ Data are shown as mean ± SE (*n* = 15). Different lowercase letters indicate significant differences among treatments by the LSD test (*P* < 0.05).

The inhibitory activity of 2-undecanol on eggs of *M. incognita* was tested. As shown in Fig. 1E, 2-undecanol can significantly inhibit egg hatching of nematodes, and its inhibitory ability is continuously enhanced with the increase of 2-undecanol concentration. The inhibition rate of egg hatching was 23.8% when the concentration of 2-undecanol was 10 mg/L, and 52.4% when the concentration of 2-undecanol was 20 mg/L, 84.5% and 98.5% when the concentration of 2-undecanol was 40 mg/L and 80 mg/L, which showed no significant difference, indicating that the inhibition rate of egg hatching reached a high level and tended to be flat.

**Fig 2:**
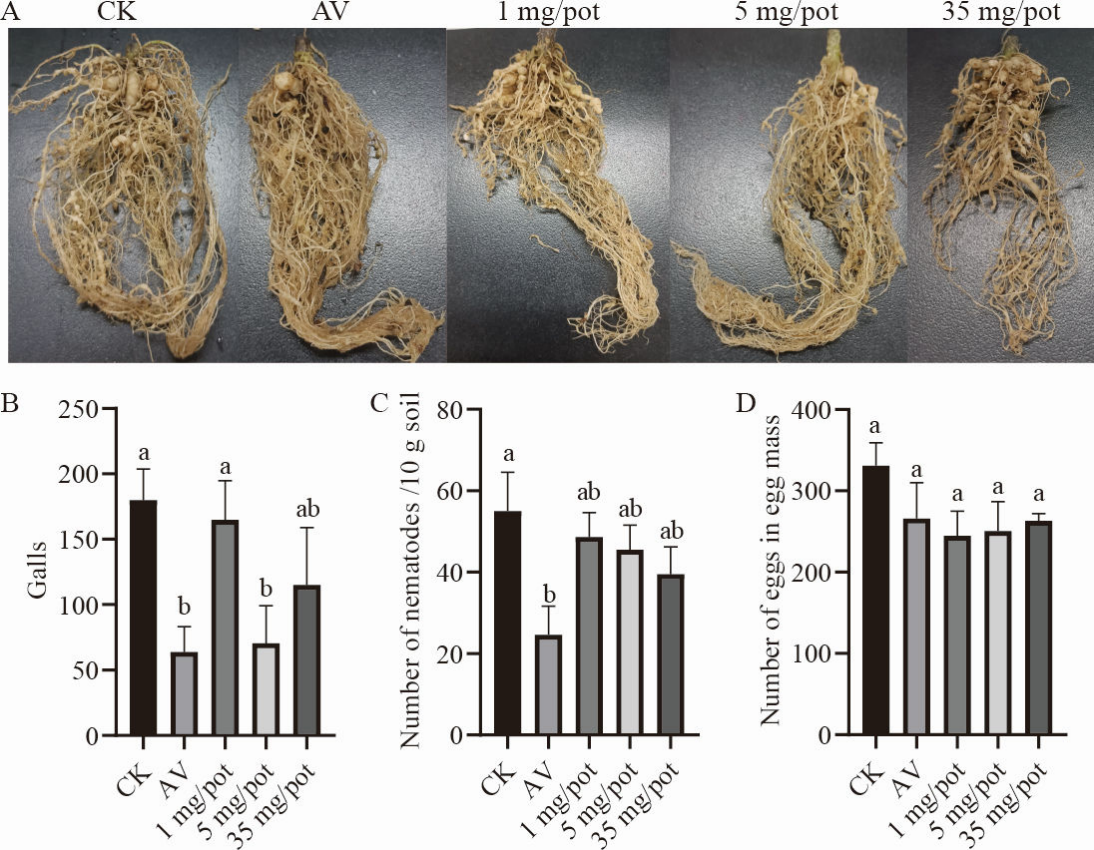
The results of the pot experiment to evaluate the effects of 2-undecanol on infection and propagation of *M. incognita*. (**A**) The pictures of tomato roots in different treated groups. Gall numbers (**B**), nematode numbers in 10 g soil (**C**), egg numbers in egg mass (**D**) under different treatments. Data are shown as mean ± SE. Different lowercase letters indicate significant differences among treatments by the LSD test (*P* < 0.05). CK: ethanol; AV: 1.8% avermectin (1 mg/pot).

### 2-Undecanol could effectively inhibit *M. incognita* from infecting tomato roots

In order to study the effects of 2-undecanol on *M. incognita* infestation and propagation, tomato plants were used for pot experiments, and the results were shown in Fig. 2. The negative control plants had large and abundant galls, while the positive control plants had fewer galls and developed roots (Fig. 2A). Compared with the negative control group, the roots of the plants in the 2-undecanol treated group were also relatively healthy. Compared with the 1 mg/pot and 35 mg/pot treated groups, the roots of the plants in the 5 mg/pot treated group had more fibrous roots and developed roots, lower in gall index, which were consistent with the results in Fig. 2B. After the application of 2-undecanol, the gall numbers of plants reduced. The control effect was only 8.4% at the dose of 1 mg/pot. When the dose was increased to 5 mg/pot, the control effect was enhanced to 60.8%, which was significantly different from the negative control effect and close to that of avermectin (64.5%). However, when the dose was increased to 35 mg/pot, the gall numbers did not decrease compared with 5 mg/pot, and even the control effect was worse than 5 mg/pot, which was 36.0%. Therefore, among these doses of 2-undecanol applied in the pot experiment, the dose of 5 mg/pot had the best control effect on nematode infection.

In order to study the effect of 2-undecanol on nematode reproduction, the density of nematodes in soil and the number of eggs in egg mass were measured. It was found that the number of nematodes in soil and eggs in egg mass did not significantly decrease after the application of 2-undecanol, as shown in Fig. 2C and D.

The effects of 2-undecanol on tomato plant growth are shown in Table 3. When the dose of 2-undecanol was 1 mg/pot and 5 mg/pot, there was no significant difference in plant height, stem thickness, above-ground fresh weight, and below-ground fresh weight, indicating that 2-undecanol at these doses had no effect on plant growth. When the dose of 2-undecanol was 35 mg/pot, the fresh weight above ground and below ground of the plants was significantly reduced compared with the negative control, and the plant height of the tomato plants was also reduced by 29.1% compared with the negative control, indicating that a high dose of 2-undecanol applied to the plant roots would have a negative effect on the growth of the plants.

**TABLE 3:** Effect of 2-undecanol on the growth of tomato^*a*^

Treatment	Plant height (cm)	Stem thickness (mm)	Above-ground fresh weight (g)	Below-ground fresh weight (g)
CK	40.50 ± 6.41^bc^	18.00 ± 0.71^b^	19.14 ± 5.00^b^	6.42 ± 1.23^b^
AV	50.02 ± 3.81^b^	18.00 ± 0.89^b^	20.49 ± 2.93^b^	5.67 ± 0.81^bc^
1 mg/pot	39.30 ± 4.69^bc^	16.00 ± 4.85^b^	13.18 ± 3.03^bc^	4.71 ± 0.56^bc^
5 mg/pot	44.03 ± 7.40^bc^	17.75 ± 1.32^b^	16.20 ± 3.96^bc^	4.45 ± 0.37^bc^
35 mg/pot	28.66 ± 3.21^c^	15.80 ± 0.80^b^	7.36 ± 1.14^c^	3.35 ± 0.38^c^

^*a*^ Data are shown as mean ± SE (*n* ≥ 4). Different lowercase letters indicate significant differences among treatments by the LSD test (*P* < 0.05).

### 2-Undecanol could induce plant roots to produce nematicidal metabolites

**Fig 3:**
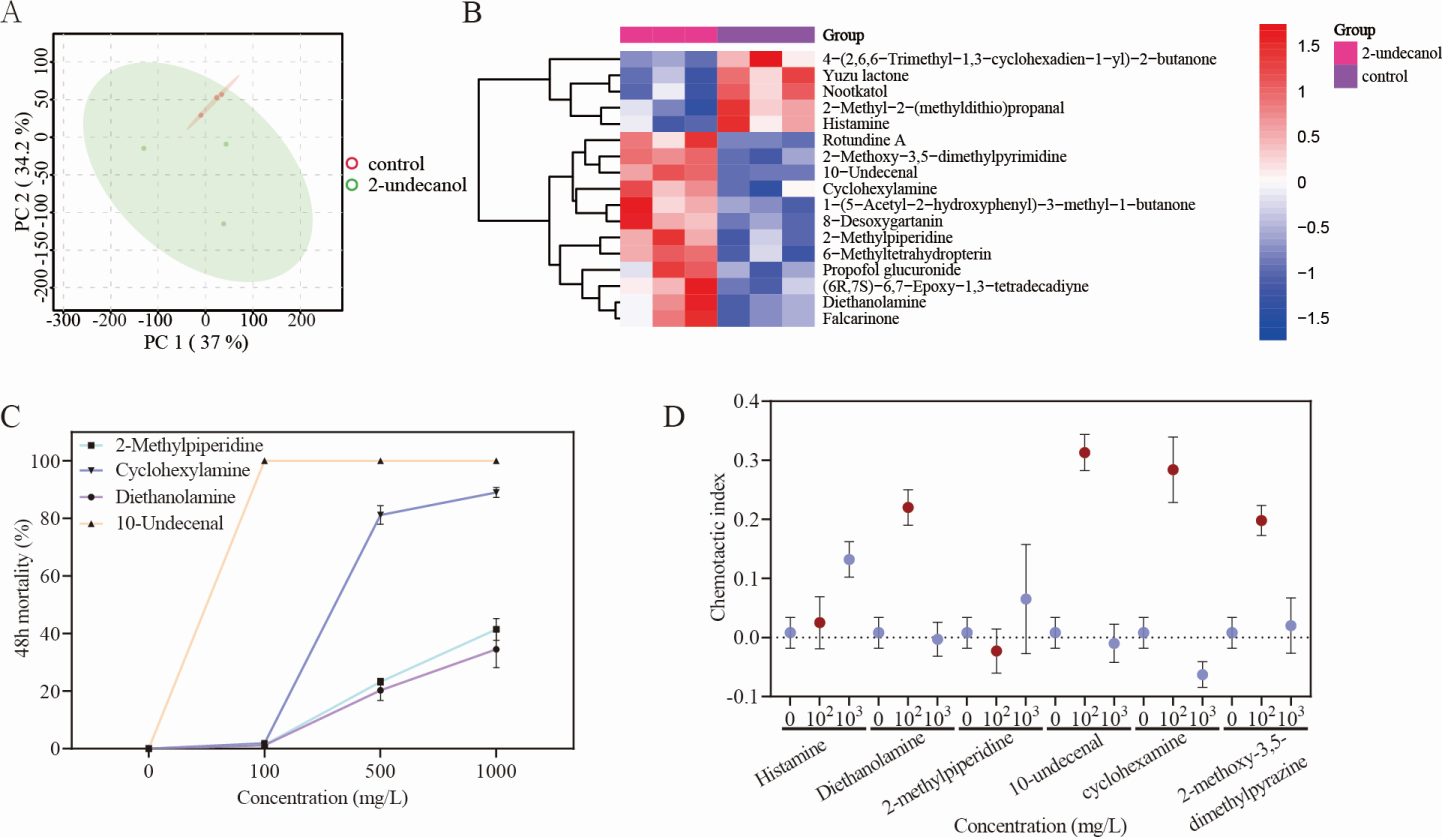
The results of non-targeted metabolome detection and effects of differential metabolites on *M. incognita in vitro*. (**A**) PCA score plot of samples. Two dots of different colors represent different groupings, where the closer the dots are, the more similar the type and content of metabolites in the sample. The dispersion of sample data is small and distributed in the ellipse of a 95% confidence interval, indicating that the analysis results are accurate. (**B**) Heatmap of hierarchical clustering analysis for the group. The horizontal coordinate represents different experimental groups, the vertical coordinate represents the differential metabolite of comparison, and the color blocks at different positions represent the relative expression of the corresponding metabolite, red indicates the high expression of the substance, and blue indicates the low expression of the substance. (**C**) Curves of contact activity against J2s of *M. incognita* immersed in solutions of 2-methylpiperidine, diethanolamine, cyclohexylamine, and 10-undecenal. (**D**) *M. incognita* chemotactic response to compounds. The data are shown as the mean ± SE (*n* ≥ 5).

According to the results in the previous chapters, 2-undecanol with the dose of 5 mg/pot was finally selected for the tomato plant pot experiment, and root exudates of treated and control plants were collected after 14 days of application of samples, and non-targeted metabolome detection was conducted to analyze their main differential metabolites. The 43,645 ion peaks extracted were summarized and sorted into a total of 43,639 ion peaks, including positive and negative ions. Principal component analysis (PCA) was performed on all the extracted ion peaks (Fig. 3A). There was a certain separation trend between the treated and control samples. The results showed that the root exudates of tomato plants were changed by the application of 2-undecanol. In order to further explore the changes of tomato root exudates and select typical differential metabolites, orthogonal partial least-squares discriminant analysis (OPLS-DA) was used to analyze the metabolome data (Fig. S1A and B). The results showed that there were significant differences between the treated and control samples, which proved that the root exudates of plants treated with 2-undecanol had significant changes.

The secondary mass spectrum information and molecular weight of metabolites were compared in the database, and a total of 17 metabolites with the most significant difference were selected according to the parameter variable importance projection of the OPLS-DA model (>1, *P* < 0.05) (Table S1). In order to find the differential metabolites clearly, cluster analysis was carried out (Fig. 3B).

In order to detect the antagonistic activity of significantly different metabolites against RKNs *in vitro*, the contact activity and chemotactic activity of the selected metabolites against *M. incognita* were detected. Among the purchased metabolites, diethanolamine, 2-methylpiperidine, 2-methoxy-3,5-dimethylpyrazine, 10-undecenal, and cyclohexylamine were up-regulated compared with the control group, and histamine was down-regulated. As shown in Fig. 3C, among the up-regulated metabolites, diethanolamine, 2-methylpiperidine, 10-undecenal, and cyclohexylamine showed great contact activity against *M. incognita*, among which 10-undecenal showed the strongest activity and completely killed nematodes at the dose of 100 mg/L for 24 h. It is also noteworthy that 10-undecenal was the most up-regulated among all the significant differential metabolites (Table S1). In addition to 10-undecenal, cyclohexylamine also had great contact activity. The mortality rate of nematodes could reach more than 90% at a concentration of 1,000 mg/L for 48 h. After treatment with 1,000 mg/L 2-methylpiperidine and diethanolamine for 48 h, nearly half of the nematodes were killed. However, treatment with 1,000 mg/L 2-methoxy-3,5-dimethylpyrazine for 48 h resulted in a mortality of less than 5% (results not listed), hence no contact nematicidal activity. Moreover, the four compounds (shown in Fig. 3C) had an additive effect on the contact activity against *M. incognita* (Fig. S2). The results showed that the up-regulated 10-undecenal, cycloheximide, diethanolamine, and 2-methylpiperidine all had nematicidal activity, while the down-regulated histamine had no nematicidal activity compared with the control group.

**Fig 4:**
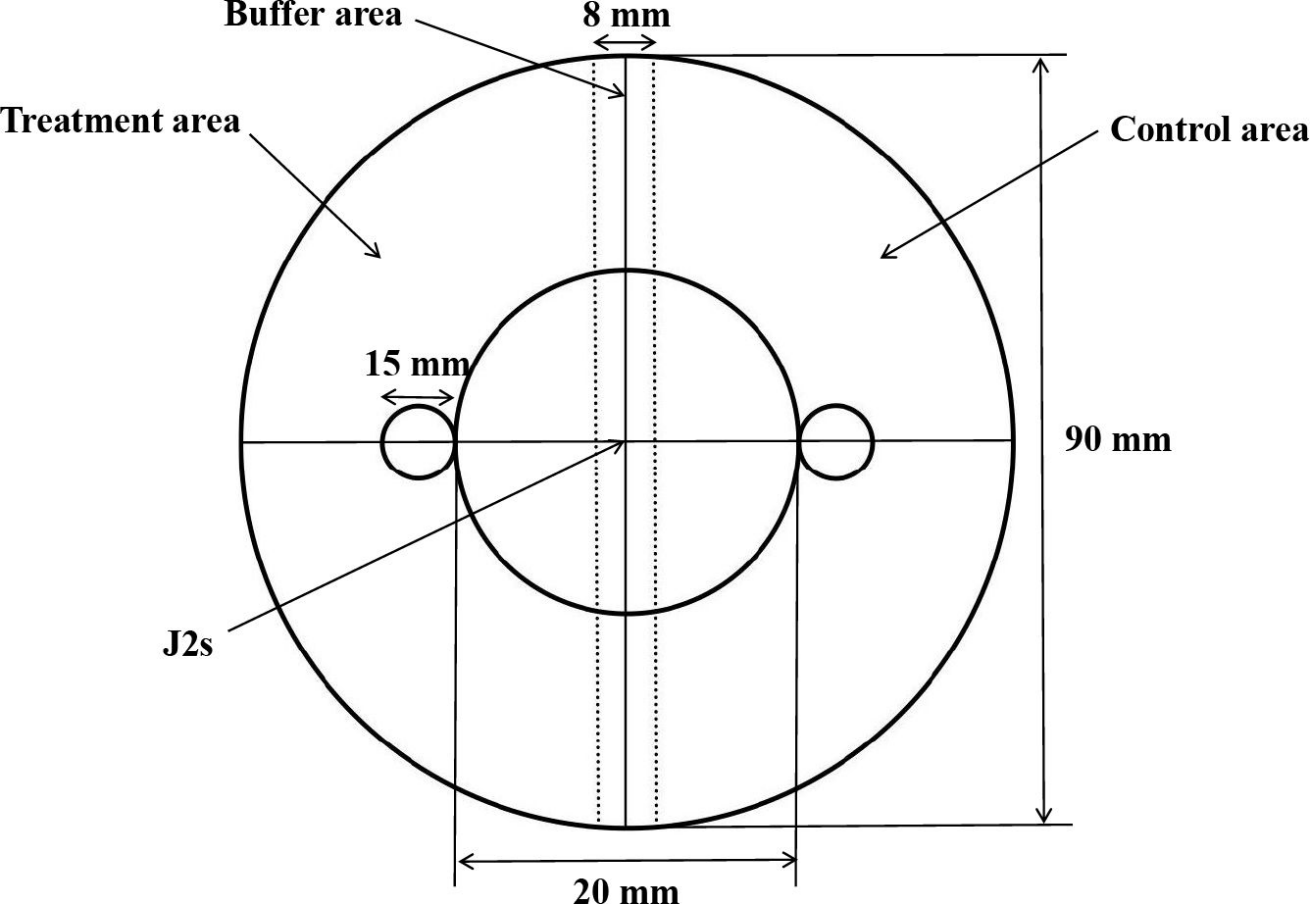
The diagram of the chemotaxis experiment.

In order to test the chemotactic activity of significant differential metabolites to *M. incognita*, the chemotaxis model (Fig. 4) was used to identify six differential metabolites. The substances were found to have attractant activity to *M. incognita*, as shown in Fig. 3D. Diethanolamine, 2-methoxy-3,5-dimethylpyrazine, 10-undecenal, and cyclohexylamine all showed attractant activities to *M. incognita* at the dose of 100 mg/L, but the chemotaxis to these substances was unstable at the dose of 1,000 mg/L. 2-methylpiperidine at the doses of 100 and 1,000 mg/L had no stable chemotactic activity to *M. incognita*. Histamine showed attractant activity to *M. incognita* at the dose of 1,000 mg/L and did not stably attract or avoid nematodes at the dose of 100 mg/L.

## DISCUSSION

In our previous work, the fermentation supernatant of *Paenibacillus polymyxa* KM2501-1 has been found to have the activity of attracting and killing *M. incognita*, and it can kill the nematode by producing VOCs. Among them, 2-undecanol exhibits potent contact and fumigation nematicidal activity against *M. incognita* (33), but other biological activities of 2-undecanol on *M. incognita* have not been studied. 2-Undecanol showed attractant activity to *M. incognita* (Table 1). However, our previous studies have found that the chemotaxis response is variable (33). Compared to a broader range of concentrations used previously, the test concentrations selected in this study showed attractant activity. Future research will further test whether other concentrations of 2-undecanol are attractants to *M. incognita*. The frequency of head thrashing and body bending is often used to evaluate the toxicity of drugs on the locomotion behavior of nematodes (36). In this study, the movement of nematodes was slowed down after treatment with 2-undecanol, and the frequency of head thrashing and body bending of nematodes decreased (Table 2). Toxicity to larvae and inhibition of egg hatching are important indicators for assessing the nematicidal potential of drugs (37). The results also showed that 2-undecanol could inhibit the egg hatching of *M. incognita* (Fig. 1E). Both 2-undecanol and its derivatives can inhibit the growth of *Bacillus subtilis* and mould *Aspergillus niger* (38). Our previous study identified the nematicidal activity of 2-undecanol against J2s of *M. incognita* (33). However, its inhibitory activity on egg hatching was studied for the first time. In conclusion, 2-undecanol not only exhibits nematicidal activity against juveniles but also shows inhibitory activity on egg hatching. All the above experiments were the activity tests of 2-undecanol against *M. incognita in vitro*, but the potential of 2-undecanol as a nematicide needs to be evaluated by pot experiments to explore its ability to affect nematode parasitism and reproduction on the plant.

The gall index of plant roots and the number of eggs in egg mass can be used to assess nematode infestation and reproduction on plants (39). In this study, different doses of 2-undecanol application reduced the gall index of tomato roots, but the control effect of nematode density in soil was not significant. It suggested that 2-undecanol could effectively inhibit the infestation of *M. incognita* in tomato roots, rather than directly reducing the number of nematodes in the soil. In addition, according to the growth status of plants after application of 2-undecanol, it was found that low doses of 2-undecanol had no effect on the growth and development of the plants, while 35 mg/pot of 2-undecanol had an inhibitory effect on the growth of the plants. Some compounds have been reported to inhibit plant growth at high doses (40). For example, substances such as ferulic acid inhibit plant growth by inhibiting the absorption of water and nutrients from the roots (41). The reason why the high dose of 2-undecanol showed an inhibitory effect on the growth of tomato plants may be that the activity of the root system was affected. This led to the inhibition of nutrient absorption, resulting in poor development, reduced immune activity, decreased ability to defend against diseases, and increased susceptibility to nematode infection. According to the results of the gall index and the growth status of plants, the best control effect was obtained at the dose of 5 mg/pot, similar to that of the positive control avermectin, which may be due to the multiple modes of function of 2-undecanol. After the application of 2-undecanol, in addition to the contact activity, it may also produce multiple modes such as fumigation, attraction, locomotion inhibition, and egg hatching inhibition to control nematodes and reduce the infection of nematodes. It can produce better control effects and reduce the cost of nematode control at a lower dose. During plant growth, roots secrete a large number of substances, many of which can affect nematodes, or lure nematodes to migrate to roots, or drive nematodes away from plant roots, or reduce the motility of nematodes so that they cannot migrate to the host roots or cannot infect the host, and even some secretions can kill nematodes (42). The glycosides and flavonoids in root exudates could affect the behavior of the *M. incognita* J2s. The chemicals in root exudates of tomato and rice could inhibit the oral needle puncture and movement of the nematode, thereby reducing the infection (43). Combined with the above results, it was concluded that the application of 2-undecanol had an effect on tomato roots, leading to a change in the composition of root exudate and thus reducing nematode infestation.

Plants release a large number of substances into the soil through their roots, which directly or indirectly affect soil organisms (44). Numerous studies have recently identified a large number of compounds with antimicrobial activity to protect plants directly from pathogen invasion (45), but relatively little has been studied on RKNs. According to the pot experiment results, 2-undecanol can effectively inhibit *M. incognita* infestation in tomato roots, and root exudates, for which attractants for PPNs are included, usually play an important role in resisting RKN infestation in plant roots (46, 47). Therefore, the application of 2-undecanol may have an effect on the root exudates of tomato. The composition and abundance of root exudates of tomato in the control and treated (5 mg/pot) groups were compared based on non-targeted metabolomics analysis. The results showed that there were significant changes in tomato root exudates (Fig. 3A). Notably, 2-undecanol was not detected in either the untreated extracts or the treated extracts. The volatile nature of 2-undecanol may have led to significant evaporation, resulting in minimal residual quantities in soil at the time of sampling. Moreover, the undetectability of 2-undecanol may result from its low solubility in water (extraction phase) (48). It was found that differential metabolites, 10-undecenal and cyclohexylamine, had great contact activity, and 10-undecenal, which also had attractant activity at the same concentration, was the best (Fig. 3C and D). The structure of 10-undecenal is similar to that of 2-undecanol, and it may be produced through an analogous reaction. 10-Undecenal may be a metabolite produced by the tomato. Detection of volatile compounds produced by spiny coriander revealed that 10-undecenal was one of the major components (49). Therefore, 10-undecenal, probably being induced by 2-undecanol, can be produced by tomato roots as a potential attractant. In addition to 10-undecenal, diethanolamine, 2-methoxy-3,5-dimethylpyrazine, and cyclohexylamine all had attractant activity to *M. incognita* at low concentration while unstable at high concentration (Fig. 3D). Nematodes could be attracted to certain compounds at low concentrations but repelled at high concentrations (50). Diethanolamine is a lipid substance in plants, which can be linked with fatty acids by amide bonds to form N-acylethanolamine, which participates in the signal transduction of plant disease resistance and enhances the defense ability of plants against diseases and pests (51–53). The increase of diethanolamine in tomato root exudates after 2-undecanol treatment might be related to the resistance of plants against *M. incognita*. The metabolic pathway of plant root exudates includes primary and secondary metabolism (54). The primary metabolism provides nutrients and energy for plant growth and development, while the secondary metabolism does not directly participate in plant growth activities but occurs when plants establish systemic resistance when subjected to stress (44). In this study, KEGG pathway analysis (Fig. S1C) revealed that pathways related to secondary metabolite biosynthesis were significantly altered, which were probably involved in the systemic resistance of plants. The results showed that 2-undecanol could affect the metabolites of tomato roots and induce the production of nematicidal substances with attractant activity or even systemic resistance to reduce nematode infestation.

In this study, we investigated the effects of 2-undecanol, a volatile metabolite of *P. polymyxa* KM2501-1, on the whole development stage of RKNs and found that 2-undecanol had multiple control modes against *M. incognita*, which provided a new strategy for the comprehensive control of root-knot nematodes. Through metabolomics analysis of the composition and abundance of tomato root exudates and testing the inhibitory activity of the differential metabolites against *M. incognita in vitro*, we established a new mechanism of 2-undecanol against nematodes by inducing tomato plants to secrete root exudates with attractant and nematicidal activity, which laid a theoretical foundation for the development of 2-undecanol as a new nematicidal agent. Not only 2-undecanol, but also the differential metabolites with nematicidal activity against *M. incognita* can serve as effective ingredients for future nematicides. Further studies are needed to investigate whether 2-undecanol can induce plant immunity or regulate the microbial community of host roots to reduce nematode infestation. In terms of microbial control, the synthetic biology-based modification of *P. polymyxa* KM2501-1 to enhance its production of 2-undecanol may enhance its efficacy against nematodes.

## MATERIALS AND METHODS

### Plant and nematode strains

Seeds of the tomato cultivar (*Solanum lycopersicum*) “Zhongshu 4,” nematode susceptible species, were purchased locally (Wuchang Shenniu Seedling business, Wuhan). The seeds were sown in the plastic seedling trays containing nutrient soil in a greenhouse maintained at (25°C ± 1°C). The (25 ± 5)-day-old seedlings were transplanted into plastic pots (cm: 13 × 13 × 11) containing 1 kg of sand and a nutrient soil mixture (1:1).

*M. incognita* was obtained from infected roots of tomato. Egg masses of *M. incognita* were peeled from infected roots with needles, washed, and hatched in 24-well culture plates with sterile water at 20°C. Freshly hatched J2s were collected and used for experiments.

### Nematicidal activity of 2-undecanol *in vitro*

To evaluate the nematicidal activity of 2-undecanol, different concentrations of 2-undecanol (dissolved in ethanol as a mother solution) were prepared for contact (10, 30, 50, 70, and 90 mg/L) and fumigation (50, 100, 200, and 400 mg/L) activity experiments in 96-well plates. Dissolvent served as the negative control. Contact: 120 µL of different concentrations of 2-undecanol solution and 30–50 J2s were added to each well. Plates were covered with plastic lids at 20°C for 48 h. Fumigation: in the middle of the 96-well plate, 200 µL of different concentrations of 2-undecanol solution was added, then 120 µL of sterile water and about 100 J2s were added to four adjacent wells. Rigid and immobile nematodes were considered dead, and the mortality rate of nematodes was measured under an inverted microscope. Four replicates were set for each treatment. The nematode mortality rates have been corrected by eliminating the natural death in the negative control (ethanol) according to the Schneider-Orelli formula (55): relative mortality rate = (mortality rate of treated group − mortality rate of control group)/(1 − mortality rate of control group) × 100%.

To assess the chemotactic activity of 2-undecanol on *M. incognita*, the bottom of each disposable petri dish was marked (Fig. 4). Subsequently, a 2% (wt/vol) agar solution was poured into each plate under sterile conditions. One filter paper (diameter 1.5 cm) was placed at each marked point, and 30 µL of 2-undecanol in different concentrations (100, 1,000, and 10,000 mg/L) was added to one filter paper, and ethanol was added to the other filter paper, so that the plate was divided into two areas (treatment area and control area). About 200 J2s were added to the center of the plate. Plates were maintained in the dark at 20°C for 8 h. The number of nematodes in the treatment (*n*) and control area (*N*) was recorded under an inverted microscope, and the C.I. was calculated, [(*n − N*)/(*n + N*)].

In order to determine the effect of 2-undecanol on motor behavior of *M. incognita*, in a 24-well plate, 200 J2s were added to each well and incubated at 20°C with 1 mL of 2-undecanol solution (0, 10, 20, and 40 mg/L) for 24 h, then transferred to sterile water for 20 min to recover. The nematodes were observed under the VHX6000 super-resolution microscope to record the frequency of head thrash (the times of the nematode’s sinusoidal movement in 20 s) and the body bend (the times of the nematode’s body bending in 1 min).

To test the inhibition of 2-undecanol on egg hatching of *M. incognita*, the egg masses were split with NaClO (1:50), and egg suspension was obtained after washing with sterile water. In 96-well plates, 120 µL of 2-undecanol solution (0, 10, 20, 40, and 80 mg/L) and 500–1,000 eggs were added to each well and incubated at 20°C for 7 days. The number of hatched nematodes was observed and recorded under the inverted microscope. The inhibition rate of hatching was determined as follows: hatching inhibition rate = (hatching rate of control group − hatching rate of treated group)/hatching rate of control group × 100%.

### Pot experiment for the analysis of 2-undecanol to control *M. incognita*

A pot experiment was conducted to evaluate the effects of 2-undecanol on infection and propagation of *M. incognita*. Ethanol served as the negative control, and 1.8% avermectin (amounting to 1 mg/pot) served as the positive control. About 3,000 J2s were inoculated uniformly around the seedlings. After inoculation with J2s, the samples were added to tomato roots (2-undecanol was dissolved in 0.5 mL ethanol) at the final doses of 1 mg/pot (the dose of avermectin treatment), 5 mg/pot (intermediate dose), and 35 mg/pot (LC_50_ of 2-undecanol *in vitro*). Each treatment was performed with five replications. The tomato plants were harvested 60 days after inoculation with J2s, and the growth indexes (plant height, stem thickness, above ground fresh weight, and below ground fresh weight) of plants were measured, and the number of galls, nematodes in 10 g soil, and eggs in egg mass were counted to evaluate the control effect.

### Collection of root exudate and metabolomics analysis

To determine the effect of 2-undecanol on root exudates of tomato plants, we cultured tomato plants exerted by *M. incognita* treated with 2-undecanol (5 mg/pot), as well as ethanol served as a control, then collected their root exudates 2 weeks after 2-undecanol application, identified and screened the root exudate components. The tomato plants were carefully pulled out, the soil covering the root surface was washed off, then the plants were put into a conical flask, the roots were completely submerged in 50 mL ddH_2_O, and the plants were put into an incubator at 20°C, and cultured for 48 h according to the optical law of 14 h of light and 10 h of darkness. The obtained root secretion was filtered to remove impurities and freeze-dried for 48 h until the samples were completely powdered and stored at −80°C. Extraction and analysis of tomato root exudates were carried out by Shanghai Biotree Biomedical Technology Co., LTD.

### Nematicidal activities of differential metabolites

According to the results of non-targeted metabolomics analysis of tomato root exudates, typical differential metabolites were screened, and the contact activity and chemotactic activity of differential compounds against *M. incognita* were tested *in vitro*. Diethanolamine (≥99.7%, CAS: 111-42-2), 2-methylpiperidine (>99.0%, CAS: 109-05-7), 2-methoxy-3,5-dimethylpyrimidine (>95.0%, CAS: 92508-08-2), 10-undecenal (>97.0%, CAS: 112-45-8), cyclohexylamine (>99.5%, CAS: 108-91-8), histamine (>96.0%, CAS: 51-45-6) were purchased from Aladdin Reagent (Shanghai) Co., LTD.

### Statistical analysis

All data were analyzed by using SPSS Software version 26.0, and are shown as the mean ± standard error (*n* ≥ 3). One-way analysis of variance and multiple comparison (LSD Duncan) were employed to test for significant differences between treatments. Different lowercase letters indicate significant difference (*P* < 0.05) among treatments.

## SUPPLEMENTAL MATERIAL

Supplemental material spectrum.03062-24-s0001.docx Fig. S1 and S2; Table S1.

ASM does not own the copyrights to Supplemental Material that may be linked to, or accessed through, an article. The authors have granted ASM a non-exclusive, world-wide license to publish the Supplemental Material files. Please contact the corresponding author directly for reuse.
