# Supplementary material for: Efficacy of 2-undecanol produced by Paenibacillus polymyxa KM2501-1 in controlling Meloidogyne incognita
Source: Microbiol Spectr. 2025 Jun 26;13(8):e03062-24. doi: 10.1128/spectrum.03062-24 (PMC12323671; doi:10.1128/spectrum.03062-24)
Supplement: Supplemental material — Fig. S1 and S2; Table S1. [file spectrum.03062-24-s0001.docx]

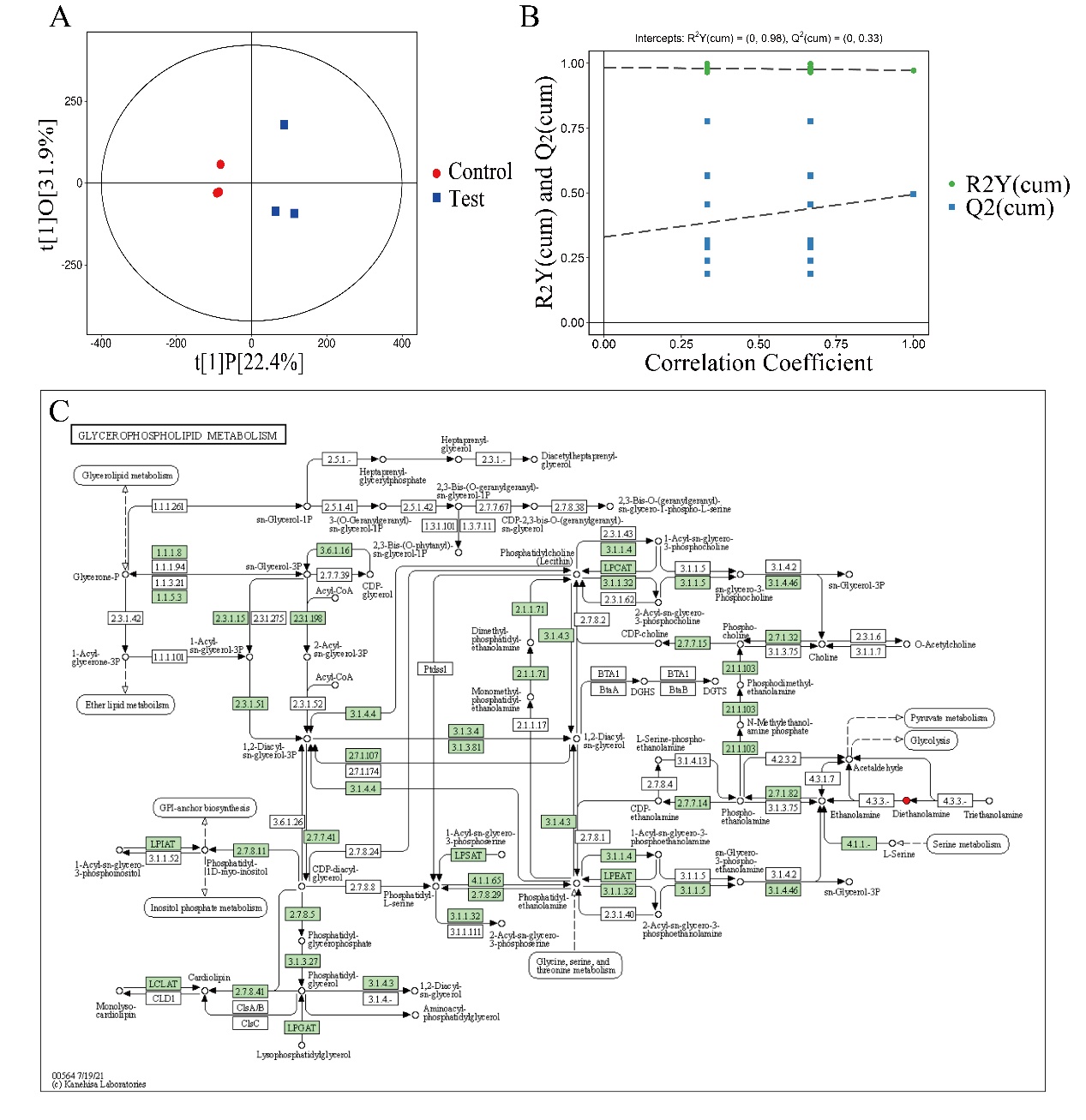


**FIG. S1.** Metabolomics analysis. (A) Score plot of OPLS - DA model. In order to increase the accuracy of OPLS-DA screening for differential metabolites, OPLS-DA modeling analysis was carried out by software, and the modeling quality of principal components was checked by 7-fold cross-validation to evaluate the effectiveness. (B) Model validation. The parameters R^2^Y (the model's interpretation of Y variable) and Q^2^ (the model's predictability) are 0.973 and 0.494, respectively, which indicates that the model conforms to the real situation of the sample data and has good stability. (C) KEGG map of glycerophospholipid metabolism.

**TABLE S1** Identification of Differential metabolites.

| ID | MS2-name | m/z | Significant | Fold change |
| --- | --- | --- | --- | --- |
| 67 | Diethanolamine | 74.096 | UP | 1.135 |
| 235 | Rotundine A | 232.169 | UP | 5.896 |
| 248 | Propofol glucuronide | 335.178 | UP | 3.917 |
| 279 | 2-Methylpiperidine | 100.112 | UP | 1.619 |
| 332 | Falcarinone | 241.155 | UP | 3.889 |
| 457 | 2-Methoxy-3,5-dimethylpyrimidine | 139.159 | UP | 4.536 |
| 638 | 4-(2,6,6-Trimethyl-1,3-cyclohexadien-1-yl)-2-butanone | 193.159 | DOWN | 0.956 |
| 709 | 10-Undecenal | 169.158 | UP | 14.627 |
| 826 | 2-Methyl-2-(methyldithio)propanal | 151.024 | DOWN | 0.658 |
| 865 | Histamine | 112.087 | DOWN | 0.438 |
| 914 | 1-(5-Acetyl-2-hydroxyphenyl)-3-methyl-1-butanone | 221.117 | UP | 3.376 |
| 935 | Yuzu lactone | 197.153 | DOWN | 0.401 |
| 970 | 6-Methyltetrahydropterin | 182.103 | UP | 1.452 |
| 1019 | Cyclohexylamine | 100.112 | UP | 1.822 |
| 1132 | 8-Desoxygartanin | 381.176 | UP | 2.883 |
| 1168 | (6R,7S)-6,7-Epoxy-1,3-tetradecadiyne | 205.159 | UP | 3.281 |
| 1240 | Nootkatol | 221.190 | DOWN | 0.922 |


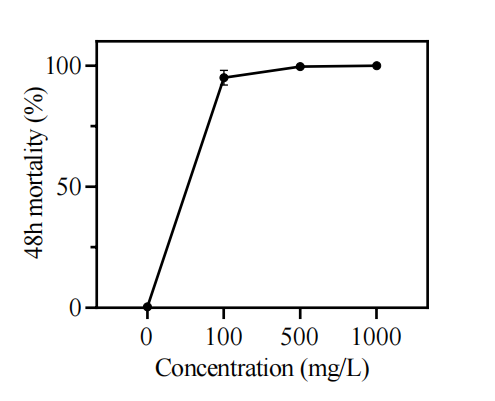


**FIG. S2.** Contact nematicidal activity against J2s of *M. incognita* immersed in mixed solution of 2-methylpiperidine, diethanolamine, cyclohexylamine and 10-undecenal.
